# Evaluation and costs of volunteer telephone cessation follow-up counseling for Veteran smokers discharged from inpatient units: a quasi-experimental, mixed methods study

**DOI:** 10.1186/s12971-015-0028-9

**Published:** 2015-02-05

**Authors:** Sonia A Duffy, Lee A Ewing, Samantha A Louzon, David L Ronis, Neil Jordan, Molly Harrod

**Affiliations:** Ohio State University, College of Nursing, Columbus, USA; Ann Arbor VA Center for Clinical Management Research, Health Services Research and Development, P.O. Box 130170, Ann Arbor, MI 48113-0170 USA; University of Michigan, Department of Psychiatry, Ann Arbor, USA; University of Michigan, School of Nursing, Ann Arbor, USA; Center of Innovation for Complex Chronic Healthcare, Hines VA Hospital, Hines, USA; Center for Healthcare Studies and Departments of Psychiatry & Behavioral Sciences and Preventive Medicine, Northwestern University, Feinberg School of Medicine, Chicago, USA

**Keywords:** Tobacco cessation, Smoking cessation, Peer support, Telephone calls, Veterans

## Abstract

**Background:**

The Reach, Effectiveness, Adoption, Implementation, and Maintenance (RE-AIM) framework was used to evaluate the volunteer telephone smoking cessation counseling follow-up program implemented as part of the inpatient Tobacco Tactics intervention in a Veterans Affairs (VA) hospital.

**Methods:**

This was a quasi-experimental, mixed methods design that collected data through electronic medical records (EMR), observations of telephone smoking cessation counseling calls, interviews with staff and Veterans involved in the program, and intervention costs.

**Results:**

*Reach*: Of the 131 Veterans referred to the smoking cessation telephone follow-up program, 19% were reached 0–1 times, while 81% were reached 2–4 times. *Effectiveness*: Seven-day point-prevalence 60-day quit rates (abstracted from the EMR) for those who were reached 2–4 times were 26%, compared to 8% among those who were reached 0–1 times (p = 0.06). Sixty-day 24-hour point-prevalence quit rates were 33% for those reached 2–4 times, compared to 4% of those reached 0–1 times (p < 0.01). *Adoption and Implementation:* The volunteers correctly followed protocol and were enthusiastic about performing the calls. Veterans who were interviewed reported positive comments about the calls. The cost to the hospital was $21 per participating Veteran, and the cost per quit was $92. *Maintenance*: There was short-term maintenance (about 1 year), but the program was not sustainable long term.

**Conclusions:**

Quit rates were higher among those Veterans that had greater participation in the calls. Joint Commission standards for inpatient smoking with follow-up calls are voluntary, but should these standards become mandatory, there may be more motivation for VA administration to institute a hospital-based, volunteer telephone smoking cessation follow-up program.

**Trial registration:**

ClinicalTrials.Gov NCT01359371.

## Introduction

A Cochrane Review has shown that inpatient smoking cessation interventions that provide telephone follow-up are more efficacious than those without telephone follow-up [1,2] and telephone counseling has been shown to improve quit rates for Veteran smokers [3]. Recent Joint Commission standards for inpatient smoking include contact with the patient 15 to 30 days after hospital discharge regarding tobacco use status [4]. However, post-discharge telephone counseling is one of the most difficult components of inpatient cessation interventions to implement.

In a prior published study, nurses were taught to implement the inpatient Tobacco Tactics intervention [5,6]. Since peer support has been shown to be effective in improving a number of health conditions and behaviors [7-11], volunteers were trained to conduct follow-up calls. Anecdotal feedback revealed that patients liked the telephone cessation counseling and volunteers were extremely enthusiastic about having the opportunity to provide cessation follow-up calls.

Using the Reach, Effectiveness, Adoption, Implementation and Maintenance (RE-AIM) framework [12,13], an evaluation of the volunteer initiated telephone smoking cessation follow-up calls was conducted. The specific aims of this study were to: 1) determine differences in the demographics and health characteristics of those who did and did not participate in the telephone counseling program (*Reach*); 2) determine if there were differences in quit rates between those who did and did not participate in the telephone cessation counseling (*Effectiveness*); 3) evaluate the implementation of the program, barriers and facilitators to implementation, the cost, and the cost per quit (*Adoption* and *Implementation*); and 4) evaluate the sustainability of the program (*Maintenance*).

## Methods

### Design, setting, sample, and procedures

This was a quasi-experimental, mixed methods study of the volunteer telephone smoking cessation counseling component of the inpatient Tobacco Tactics intervention conducted at the Ann Arbor Veterans Affairs (VA) hospital [5]. Based on their clinical judgment, nurses gave the Tobacco Tactics intervention to any smoker they thought was physically and mentally able and willing to participate in the intervention. Once discharged from the inpatient unit, participants (N = 131) were then referred to the follow-up telephone counseling component of the intervention from June 2012 to March 2013. The sample also included 4 staff: the volunteer coordinator, the smoking cessation coordinator, and two volunteer smoking cessation counselors. Human Studies approval was received from the Ann Arbor VA Subcommittee on Human Studies - VA Institutional Review Board.

To determine the *Reach* and *Effectiveness* of the calls, data were collected from electronic medical records (EMR). To determine the *Adoption* and *Implementation* of the calls, staff were interviewed and asked about the barriers and facilitators to providing the smoking cessation telephone calls. Fifteen volunteer calls were observed and field notes were taken to determine if the components of the telephone counseling were being implemented. Volunteer-Veteran interactions were also observed. In addition, 25 of the 131 Veterans were interviewed. Veteran interviews were audio-recorded and transcribed. The costs and cost per quit from the perspective of the payer were determined. *Maintenance* was determined by the sustainability of the program once research ended.

### Intervention

Details about the inpatient Tobacco Tactics intervention have been previously published [5,6]. In brief, the inpatient Tobacco Tactics intervention conducted by staff nurses provides those interested in quitting with behavioral and pharmaceutical intervention. If the nurse indicated on the documentation template that the patient was given the Tobacco Tactics manual, the EMR was programmed to add the patient’s name and phone number to a list that was forwarded to Voluntary Services on a weekly basis. Trained volunteers initiated follow-up cessation counseling to patients at 2, 14, 21 and 60 days after in-patient discharge; three attempts were made per time point. Since volunteers cannot access or chart in the EMR, volunteer documentation was entered into the EMR by support staff.

Working with the director of Voluntary Services, never smoking or long-term non-smoking volunteers were handpicked (based on their comfort/skill level with talking on the phone and providing support) and trained to provide the telephone cessation counseling. Volunteer training consisted of: 1) participating in the 1-hour Tobacco Tactics training program; 2) viewing the video shown to patients about smoking cessation; and 3) viewing the video “Tools for Being a Helpful Peer Partner” about peer support that has been used in other studies [14]. Volunteers were given a script that covers three important aspects of providing support to smokers, namely: 1) positive reinforcement; 2) handling thoughts about smoking; and 3) strategies to cope with cravings. Volunteers were supervised making calls until the research staff were comfortable with their performance and execution of the protocol. Volunteers provided only behavioral support and referred all medical questions or unanticipated situations/crises to case managers or 911.

### Measures

#### Reach and effectiveness

The *Reach* of the telephone cessation counseling was measured by the number of contact calls in which the patient talked with volunteers (0 to 4); number of calls was dichotomized into 0–1 (n = 25) versus 2–4 (n = 106) representing those who were marginally engaged (only 5 people had 0 calls) and those who were more engaged. This dichotomization was conducted because some of the groups had small numbers and this grouping provided 80% power to detect a medium-large effect and 60% power to detect what Cohen defined as a medium sized effect [15]. See detailed power analysis below.

Patient characteristics, including demographics and comorbidities, were abstracted from the EMR. Smoking status around 60-days post-discharge (range 1 month to 5 months) was collected from EMR text fields (smoked in the past 7 days—yes/no) or 60-day volunteer documentation (smoked in the last 24 hours—yes/no). When smoking status data was missing, participants were considered to be a smoker.

#### Adoption and implementation

Volunteers were observed making calls implementing the components of the counseling (fidelity) and their interactions with Veterans (e.g., providing empathy and encouragement). Questions for the patient interviews were formulated to reflect their role and experience in the program. For example, patients who quit smoking during the counseling program were asked “What happened after they stopped calling you? Did you have any trouble continuing not to smoke?” and “Is there anything the program could have done differently that would have been more helpful to you?” Staff interview questions focused on identifying barriers and facilitators to implementation. For example, volunteers were asked “How did your training on peer support influence your counseling?” and the smoking cessation coordinator was asked “Are there any organizational barriers to implementing the program?”

The primary cost component of the intervention is the cost of labor by providers to train the volunteers and enter volunteer documentation into the EMR. These costs were estimated using VA salary and fringe benefit information obtained from the Financial Management System. Number of hours spent by the volunteers providing telephone counseling was also tracked. Other intervention costs included nominal supplies associated with training and the intervention. Because the volunteers made their telephone calls in an unused office, the cost of space was not included. Recruitment and other research-related costs were excluded.

### Data analysis

Power analyses were conducted with PASS software [16] using the methods recommended by Cohen (1988). Analyses to evaluate *Reach* (Aim 1) were based on t-tests, chi-square tests, and Fisher’s exact tests comparing survey respondents from the samples who used (n = 106) or did not use (n = 25) the telephone counseling. This sample size provides 83% power to detect a medium-large sized difference between the means or proportions in the two groups (based on Cohen's definitions of effect sizes). Power for analysis assessing *Effectiveness* (Aim 2) was similar to that for *Reach* in that the same subgroups were being compared. Analyses for the other aims (Aims 3–5) were descriptive, so power analysis was not directly relevant to them*.*

#### Reach and effectiveness

Descriptive statistics (means and frequencies) were calculated for all quantitative variables. Bivariate relationships between a patient’s participation in the program and demographic and health behavior characteristics as well as bivariate relationships between participation and variables representing smoking status were calculated. T-tests for normally-distributed continuous variables and Wilcoxon Exact tests for non-normally distributed continuous variables were used to detect differences in means, and Chi-Square or Fisher’s exact tests were used to detect differences in proportions. All significance tests were two-tailed and significance was set at the alpha = 0.05 level. Quantitative statistical analyses were performed using SAS version 9.3 (SAS Institute Inc., Cary, NC).

#### Adoption and implementation

To determine mean cost of the program, intervention and implementation costs were added together to calculate total cost. Total cost was divided by the number of patients served to derive mean cost per patient, and by the number of quitters to derive mean cost per quit. Descriptive analysis of field notes and interview transcripts was performed. Two of the co-authors (LE and SL) read each field note, transcribed the notes independently, and developed codes. They then met to discuss the codes and develop overarching themes. Codes were then aggregated under their corresponding theme and documented in a codebook. Any disagreements were resolved through discussion and consensus.

## Results

### Reach

Bivariate analyses shown in Table 1 indicate that 19% of the sample was reached 0–1 times while 81% were reached 2–4 times. Those who were reached 2–4 times tended to be older than those reached only 0–1 times (p = 0.07). Patients who were reached 2–4 times were more likely to have comorbid lung disease (p < 0.01).

**Table 1:** **Predictors and outcomes of patient participation in volunteer telephone cessation counseling (n = 131)** ^**a**^

	**Number of times reached by volunteer counselor**				
**Predictor**	**0-1**		**2-4**		
	**Mean**	**SD**	**Mean**	**SD**	**p**
Age (years) (n = 131)	54.7	10.7	58.5	9.2	0.07^b^
	**N**	**%**	**N**	**%**	**p**
Total	25	19.1	106	80.9	
*Sex (n = 131)*					1.00^c^
Male	24	96.0	101	95.3	
Female	1	4.0	5	4.7	
*Race (n = 130)*					0.49^c^
Non-Hispanic white	21	84.0	94	89.5	
Other	4	16.0	11	10.5	
*Marital Status (n = 130)*					0.83^d^
Married	8	32.0	36	34.3	
Not Married	17	68.0	69	65.7	
*Education (n = 128)*					0.82^d^
High school diploma/GED or less	13	54.2	59	56.7	
Some college or more	11	45.8	45	43.3	
*Employment status (n = 130)*					0.22^d^
Employed	7	28.0	18	17.1	
Not currently employed	18	72.0	87	82.9	
*Comorbid lung disease* ^*e*^ *(n = 131)*					*<0.01* ^*c*^
Yes	2	8.0	39	36.8	
No	23	92.0	67	63.2	
*Number of comorbidities (n = 131)*					0.43^d^
0-2	7	28.0	22	20.8	
3 or more	18	72.0	84	79.3	
*Outcome*					
*Used any tobacco products in the past 7 days at 60 days post-discharge? (n = 131)*					0.06^c^
Yes	23	92.0	78	73.6	
No	2	8.0	28	26.4	
*Used any tobacco products in last 24 hours at end of 60 day volunteer calls (n = 131)*					*<0.01* ^*c*^
Yes	24	96.0	71	67.0	
No	1	4.0	35	33.0	

^a^Totals vary due to missing data.

^b^P-value from *T*-Test.

^c^P-value from Fisher’s Exact test.

^d^P-value from Chi-Square test.

^e^All other comorbidities were tested and did not significantly differ by number of times reached (cancer, heart disease, hypertension, stroke, psychiatric problems, substance abuse, diabetes and arthritis).

### Effectiveness

Seven-day point-prevalence 60-day quit rates (abstracted from the EMR) for those who were reached 2–4 times were 26% compared to 8% among those who were reached 0–1 times, tending strongly toward significance (p = 0.06) (97% follow-up rate with 4 assumed to be smokers). Sixty-day 24-hour point-prevalence quit rates (abstracted from volunteer documentation) were 33% for those reached 2–4 times compared to 4% of those reached 0–1 times (p < 0.01) (74% follow-up rate with 34 assumed to be smokers).

Sub-analyses were conducted to determine if there were differences in effectiveness by comorbidities or admitting diagnosis. Compared to those without lung disease, those with lung disease had a higher 24-hour point-prevalence quit rate at 6 months (39.02% versus 22.22%, p < 0.05), but there was no difference in 7-day point-prevalence 6-month quit rates. Those admitted for psychiatric/substance abuse disorders tended to have lower 7-day point-prevalence quit rates at 6-month follow-up compared to those who were not admitted for psychiatric/substance abuse disorders (5.56% versus 25.66%, p < 0.08), but there was no difference in 24-hour 6-month point-prevalence quit rates.

### Adoption and implementation

Over a 9 month period, two volunteers spent approximately 1.5 hours each per week making 874 calls. Of the 874 call attempts, 384 calls reached respondents where the majority of patient conversations with the volunteer counselor lasted 1–3 minutes. Observations of 15 phone calls found that volunteers offered positive encouragement and suggested tips for handling thoughts about smoking and cravings to all patients. The volunteers were empathetic and understanding, particularly to patients who had set-backs and were currently smoking more cigarettes. In two instances, per protocol, the volunteers referred the patient to his/her doctor for medical advice.

Themes from patient interviews shown in Table 2 revealed that Veterans were enthusiastic about the program (“I live here all alone and I don’t have very many people to talk to but like you know, you guys were cheering me on or vouching for me to quit” (Veteran 14); “It made me feel like you know, there’s people out there that really care about your health” (Veteran 16)). Patients liked and appreciated the support from the volunteers (n = 21), with one Veteran saying “I had it [a cigarette] in my hand, getting ready to light it up, and after talking with her [the volunteer] I put it off for 2–3 hours before I smoked it” (Veteran 22). Common suggestions for improvement include more phone calls over a longer period of time (n = 6) and better patient access to smoking cessation medications (n = 5).

**Table 2:** **Summary of themes/concepts from Veteran interviews (n = 25)**

**Theme**	**Number of patients**
*Reasons for participating in Tobacco Tactics program*	
Wanted to quit smoking or thought they should quit smoking	19
Liked the quit smoking program in the hospital, or said it was nice/good/great	9
Wanted to help someone else who was trying to quit smoking	4
*Views on phone calls*	
Calls were helpful/nice/good/great	23
Support from the volunteer was nice/good/great	21
Liked that someone cared/was interested about them/their smoking	16
Liked the number of phone calls/thought number was appropriate	14
Thought the phone calls lasted 5 minutes or more	12
Preferred more phone calls	7
Does not remember what was specifically talked about during phone calls	5
Calls put the idea of quitting back in their head again/they were a reminder	3
*What happened with smoking status after phone calls stopped?*	
No difference	11
Increased smoking	6
Decreased smoking	4
*Suggestions for improvement*	
Nothing else that the program could have done to be more helpful	18
Nothing the VA could do to improve the program	6
Increasing the number of phone calls/length of follow-up	6
Make smoking aids (e.g. patches) more accessible	5
Provide more specific information during phone calls about quitting smoking	1

The volunteer counselors expressed that they felt properly prepared for being a telephone cessation counselor and that they enjoyed counseling Veterans. According to staff, the greatest strength of the program was that it was “peer-to-peer” and Veterans “realize someone cares about their tobacco cessation” (Volunteer 2). The program gives volunteers the opportunity to participate in “more hands-on [work] with Veterans than mailings or entering survey results” (Volunteer 1). In terms of maintenance, the greatest organizational barriers to implementing the program were lack of space, a coordinator who can “own” the program, and restrictions on volunteers being able to document in the EMR.

The total cost to the Ann Arbor VA of implementing the intervention was $2,772. Based on the 131 Veterans who were successfully counseled at least one time, the mean cost per participant was $21. Based on the 30 Veterans who quit smoking for at least 7 days at 6-month follow-up, the mean cost per quitter was $92. If the value of the volunteers’ time is included in the total cost calculation ($4,410), the mean cost per quitter rises to $147. See Table 3 for a detailed description of costs.

**Table 3:** **Summary of costs for the volunteer telephone counseling program**

**Component**	**Fixed or variable**	**Perspective impact**	**Activity/Subcomponent**	**Who/Hourly rate**	**Hours spent**	**Cost**
Training volunteers	F	VA	Development of 1-hour PPT presentation	Already existed	0	$0
			Development of 30-minute peer support video	Already existed	0	$0
			Training set-up (putting together binder, room reservation, setting up PPT/video)	Coordinator $46/hr.	2	$92
			Training session: 30-minute overview	Coordinator $46/hr.	0.5	$23
			Training session: 1-hour PPT presentation	Study nurse $34/hr.	1	$34
			Training session: 30-minute peer support video	Coordinator $46/hr.	0.5	$23
			Training session: 1-hour training/observation	Coordinator $46/hr.	1	$46
Weekly meetings with volunteers	V	VA	Meet with volunteers to get started/10 minutes twice/wk. for 9 mos.	Coordinator $46/hr.	12	$552
Volunteer documentation into EMR	F, V	VA	Printing of 475 volunteer sheets	Staff Support $32/hr.	0.25	$8
			Development/integration of the volunteer documentation template	Clinical Applications Coordinator $56/hr.	2	$112
			Entering documentation from peer volunteer calls into EMR (1.5 hrs./wk.)	Staff Support $32/hr.	58.5	$1,872
Non-labor costs	F	VA	Paper for 475 volunteer sheets			$10
Overhead costs	F	VA				$0
*Subtotal without volunteer salaries*						*$2,772*
Peer volunteer time costs	V	If volunteers paid	Calling study patients (1.5 hrs./wk. for 9 mos.)	Volunteer 1 $14/hr.	58.5	$819
			Calling study patients (1.5 hrs./wk. for 9 mos.)	Volunteer 2 $14/hr.	58.5	$819
*Subtotal volunteer salaries*						*$1,638*
*Total if volunteers paid*						*$4,410*

Note: All hourly rates rounded to nearest dollar and include salaries and fringes.

### Maintenance

The volunteer telephone counseling program was initially coordinated by the research team. Then it was transferred to a staff person and was sustainable for about a year after the study ended until this person resigned. It was resurrected a second time by the research team, but we were unable to get a department to take responsibility for the program so it was discontinued. In discussions with leadership, it seemed that they felt the smoking cessation counseling could be conducted as part of a call that is made to all inpatients after discharge. On both occasions when the program was terminated, volunteers were disappointed, as they really felt that they were making a difference in patients’ lives.

## Discussion

### Reach and effectiveness

The data show that the vast majority of smokers were reached 2 to 4 times. Those reached more often were more likely to quit smoking. Those with lung disease were more likely to be reached and to quit, perhaps because they had higher motivation to quit [17]. However, those admitted for psychiatric/substance abuse tended to be less likely to quit. Research has shown that those with psychiatric and substance abuse disorders are interested in quitting [18], although quitting can be difficult among this population [19].

The greater effectiveness of the telephone cessation counseling program among those with greater participation may have been because those engaged in the calls were more motivated to quit. Moreover, the effectiveness of the telephone counseling may have been attenuated by the effectiveness of the Tobacco Tactics program as a whole [6], as all patients enrolled in this study received the Tobacco Tactics intervention during hospitalization. Without randomizing to phone calls versus no phone calls, there is really no way to determine the added effect of the telephone counseling. What we do know from this study is that those who participated more in the follow-up calls were more likely to quit. Cochrane Collaborative reviews [1,2] indicate that follow-up calls improve quit rates, which is why the calls were a component of the Tobacco Tactics intervention.

In this case the telephone counseling was conducted by fellow Veterans. Peer support has been shown to be effective in improving a number of health conditions and behaviors [7,8], and Veterans have been shown to be successful peer counselors [9-11]. Those Veterans participating in peer support programs were satisfied with the help they received [9], and the trained counselors gained the proper knowledge, skills and confidence needed to help their fellow Veterans [10]. Social support is an important component of a successful smoking cessation program as is noted in the VA/Department of Defense (DoD) Clinical Practice Guidelines for Management of Tobacco Use [20], and has been shown to increase quit rates in a number of studies [21-26].

### Adoption and implementation

Observations of the volunteer phone calls demonstrated that they were able to implement the protocol as designed and reach a high number of smokers in a short period of time. Moreover, the volunteers were very engaged in helping the Veterans quit smoking. Smoking cessation interventions in general have been shown to be cost-effective [27], and the volunteer phone call program was implemented at a relatively low cost.

The VA system has recently implemented 1-855-QUIT-VET, which is a quitline that provides tailored smoking cessation counseling to Veterans [28]. Smoking cessation counselors schedule up to four follow-up calls as needed. However, unlike the program described in this paper, 1-855-QUIT-VET does not capitalize on a face-to-face intervention implemented on inpatient units. Moreover, 1-855-QUIT-VET requires an initial contact be made by the Veteran, while our volunteer program proactively called Veterans. While cold calling is sometimes thought to be intrusive, it has been shown to increase quitline utilization [29] and to enroll a different group of smokers that may be missed by standard quitline procedures [26]. Similar to our study, older male smokers in more rural areas who showed less nicotine dependence and less motivation to quit have been shown to be more likely to be captured by cold calling [30]. Rural smokers report an overall low level of access to treatment for nicotine dependence [31] and cold calling programs may provide a cost-effective way to reach these Veteran smokers.

### Maintenance

While the program did have some short-term sustainability, there are several possible reasons that the program was not sustained long-term. Moving evidence into practice and changing provider behavior is difficult. This is in part due to the multiple demands placed on providers, who are forced to respond first to multiple mandates in the face of competing resources. Rolling the telephone cessation into broader follow-up calls made to discharged patients is one way to provide the follow-up without having a separate program, but the counseling is likely to become watered down in the face of other issues that patients may present. At this point, the Joint Commission standards for inpatient smoking, including follow-up calls, are voluntary [4]. Should these standards become mandatory, there may be more motivation for administration to institute an inpatient smoking cessation program with volunteer cessation telephone follow-up.

#### Limitations of the study

This was a quasi-experimental study and, therefore, did not have a control group. There may have been selection bias as those who benefited most from the program may have been more likely to participate. Quit rates were not cotinine-verified. The intent-to-treat analysis and small sample size may have contributed to findings that trended toward, but did not quite reach significance. Lastly, this was an evaluation of a program that was implemented within the Ann Arbor VA and may not be generalizable outside of this setting. Despite these limitations, this study demonstrated the benefits of using volunteers to make follow-up calls to inpatients that received a smoking intervention in a real world setting.

## Conclusion

Using volunteers as counselors to support Veterans in quitting smoking as a follow-up to an inpatient, nurse-delivered intervention was a novel idea with goals to minimize costs of providing smoking cessation follow-up calls as well as decrease intervention burden on nursing staff. The *Reach*, *Effectiveness*, *Adoption*, and *Implementation* was high, and while the intervention was not *Maintained* long term, it was *Maintained* short term*.* Sustainable models for following smokers that receive inpatient cessation interventions are needed to enhance quit rates.
